# Transparent Film-Type Vibrotactile Actuator Array and Its Haptic Rendering Using Beat Phenomenon

**DOI:** 10.3390/s19163490

**Published:** 2019-08-09

**Authors:** Dong-Soo Choi, Sang-Youn Kim

**Affiliations:** Advanced Technology Research Center, Interdisciplinary Program in Creative Engineering, Korea University of Technology and Education, Cheonan-si 31253, Korea

**Keywords:** vibrotactile actuator array, beat phenomenon, haptic simulation, vibration frequency, carrier frequency

## Abstract

The most important thing in a thin and soft haptic module with an electroactive polymer actuator array is to increase its vibrotactile amplitude and to create a variety of vibrotactile sensations. In this paper, we introduce a thin film-type electroactive polymer actuator array capable of stimulating two types of human mechanoreceptors simultaneously, and we present a haptic rendering method that maximizes the actuators’ vibrational force without improving the array’s haptic performance. The increase in vibrational amplitude of the soft electroactive polymer actuator array is achieved by creating a beat vibration, which is an interference pattern of two vibrations with slightly different frequencies. The textures of a target object are translated into haptic stimuli using the proposed method. We conducted qualitative and quantitative experiments to evaluate the performance of the proposed rendering method. The results showed that this method not only amplifies the vibration’s amplitude but also haptically simulates various objects’ surfaces.

## 1. Introduction

Flexible and rollable displays have been developed and commercialized as consumer electronics (such as televisions and cell phones) in recent years. Especially, a flexible and rollable mobile device is one of the most promising avenues of consumer electronics. To satisfy this demand, many efforts have been made to develop essential flexible electronics, such as flexible displays, electrodes, sensors, and batteries [1,2,3,4,5]. Meanwhile, vibrotactile actuators have become one of the critical electronic parts of mobile consumer electronic devices, because most of the devices have replaced mechanical buttons with a touch panel. Although conventional vibrotactile actuators are widely used for providing tactile feedback to the user, they are usually rigid [6,7,8,9]. Therefore, it is not easy to apply these actuators to flexible mobile consumer electronic devices. To overcome these problems, many researchers have developed flexible vibrotactile actuators using an electroactive polymer (EAP) [10,11,12,13,14,15,16,17,18,19,20].

EAPs can be categorized into two types: Ionic and electric [17]. Ionic EAPs actuate under low electric fields and have a large deformation [10,11,12]. However, ionic EAPs have a slow response time and a weak force. In addition, there is a need to prevent leakage of the ionic material. Because of these characteristics, ionic EAPs can hardly be suitable materials for a haptic actuator. To create a strong force with a fast response, electric EAPs have been studied intensively [12,13,14,15,16,17,18,19,20]. Kim et al. presented a transparent soft actuator based on graphene electrodes [13]. Lee et al. proposed a new arrayed tactile module using dielectric electroactive polymers [14]. Ganet et al. developed solid state actuators based on electroactive polymers and applied them to a mobile device [15]. Phung et al. presented a tactile display device using a dielectric electroactive polymer for stimulating human mechanoreceptors [16]. Ozsecen et al. demonstrated a dielectric EAP-based vibrotactile actuator, which can be used as a telepresence device [17]. To increase the vibrational force of the electric/dielectric EAP based actuator, a new structure in which two or more effects contribute to activation of the vibrotactile actuator at the same time is needed. Yang et al. developed a flexible hybrid tactile display consisting of an electroactive polymer (EAP) module and an electrovibration module [18]. Mun et al. fabricated a soft vibrotacitle actuator through multilayered accumulation of thin electroactive polymer (EAP) films and then developed a tactile stimulation interface applicable to wearable devices [19]. Pyo et al. proposed a robust flexible tactile actuator based on a pyramidal microstructured dielectric elastomer layer [20]. These works can create a wide range of operating frequencies to create various haptic sensations. However, the vibration generated from the soft haptic actuators based on the electric EAPs is not strong enough to convey various haptic senses to the user. Therefore, to maximize the vibration amplitude, wave superposition (which is the sum of two or more waves) can be implemented. When two or more vibrations traverse on the same surface, the amplitude of the superposed vibration will become equal to the sum of the amplitudes of each wave. However, the amplitude of the superposed vibration depends on the phase difference between the applied waves. For example, let us consider a case where two vibrations, whose amplitude and frequency are the same, are applied onto a plate. If their phase difference is zero, the amplitude of the superposed vibration will be the sum of the amplitudes of the two applied vibrations. However, when the phase difference is 180°, the amplitude of the superposed vibration will be zero. Therefore, the phase control of the two vibrations is one of the most important factors in magnifying the amplitude of the output vibration. However, it is difficult to control the phase precisely, because it is affected by various factors, such as the material of the medium, the control circuit, or the length of the electric wire.

In acoustic fields, to minimize the phase effect of two input sources, Sharapov V. et al. used beat effects to create low frequency acoustic vibration [21]. In this paper, we propose a new haptic rendering method based on the beat phenomenon to haptically simulate the surface of a target object. The beat vibration is an interference pattern of two different vibrations with slightly different frequencies. The beat vibration is created by applying two vibration signals with slightly different frequencies to a target device. Let us consider the case where two haptic actuators, which are actuated at the frequency of $f_{1}$ and $f_{2}$, respectively, are attached to the target device. If two actuators have a slightly different operating frequency, the target device vibrates at the frequency of $\left(f_{1}+f_{2}\right)/2$. At this time, the envelope frequency of the vibration (we call it beat frequency) becomes $f_{1}-f_{2}$. So, the beat wave consists of a carrier wave ($\left(f_{1}+f_{2}\right)/2$) and an envelope wave ($f_{1}-f_{2}$), whose amplitude is amplified. Therefore, the proposed rendering method increases the vibrational force of a soft haptic actuator based on the electric EAP without improving thr actuator’s hardware performance through synthesizing two or more vibrations. Furthermore, the proposed method can provide a beat wave with two frequencies to the user. The main difference between conventional rendering methods [22,23,24,25] and the proposed method is that the proposed rendering method not only increases the amplitude of the vibration force, but also stimulates two mechanoreceptors simultaneously. To demonstrate the effectiveness of the proposed rendering method, we have developed a transparent film-type actuator array based on a piezoelectric polymer, which is an electric EAP. We applied the proposed rendering method to this vibrotactile actuator array and conducted both quantitative and qualitative experiments using the system.

## 2. Transparent Film-Type Actuator Array

**Figure 1:**
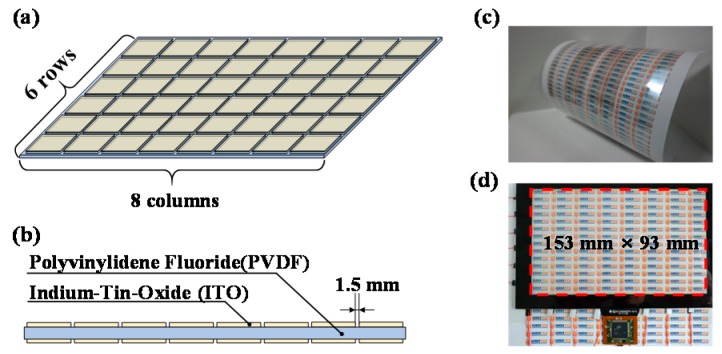
Transparent film-type vibrotactile actuator array. (**a**) Schematic of the developed actuator array and (**b**) its cross-section view. (**c**) High flexibility of the actuator array and (**d**) its optical transmittance.

Figure 1a shows the schematic of a developed thin and transparent vibrotactile actuator array of size 6 × 8 columns. The proposed actuator array was fabricated by applying indium tin oxide on both sides of a polyvinylidene fluoride (PVDF) film at a specific spacing of 1.5 mm, as shown in Figure 1b. Figure 1c shows the fabricated thin and transparent vibrotactile actuator array, which has high flexibility. Subsequently, we attached the developed vibrotactile actuator array to the rear of a touch panel, as shown in Figure 1d. The proposed actuator array, which is composed of a thin piezoelectric polymer film and transparent electrodes, fits into the touch panel, whose size is 153 × 93 × 0.8 mm^3^. PVDF was used as the vibrating film because it has good mechanical properties and large piezoelectricity [26,27]. The PVDF has a complex structure including several crystalline phases (*α*, *β*, *δ*, *γ*, and *ε*-phases) depending on the chain conformations. The *β*-phase exhibits the highest dipolar moment among all the crystalline phases [28]. This means that the relative fraction of the *β*-phase in the PVDF chain conformations highly affects the performance of the actuation. Therefore, we needed to increase the relative fraction of the *β*-phase in the PVDF to maximize the actuation performance. To transform the other phases into *β*-phase, we stretched the PVDF film mechanically at a temperature of 80 °C and obtained a thickness of 80 µm [29,30].

The PVDF is one of piezoelectric polymers having two piezoelectric effects: Direct and reverse. It generates an electric charge when an external stress (mechanical, acoustic, or thermal) is applied, which illustrates the direct piezoelectric effect, and undergoes a mechanical deformation when an electric field is applied, which illustrates the reverse piezoelectric effect. In this paper, the reverse piezoelectric effect of the PVDF was adopted to generate the mechanical vibration. When an external electric field is applied, the PVDF is stretched in its horizontal direction and compressed in its vertical direction, which is in the direction of its thickness. When the external electric field is withdrawn, the PVDF returns back to its original shape. Thus, the AC voltage makes the PVDF vibrate.

## 3. Haptic Rendering Based on the Beat Phenomenon

**Figure 2:**
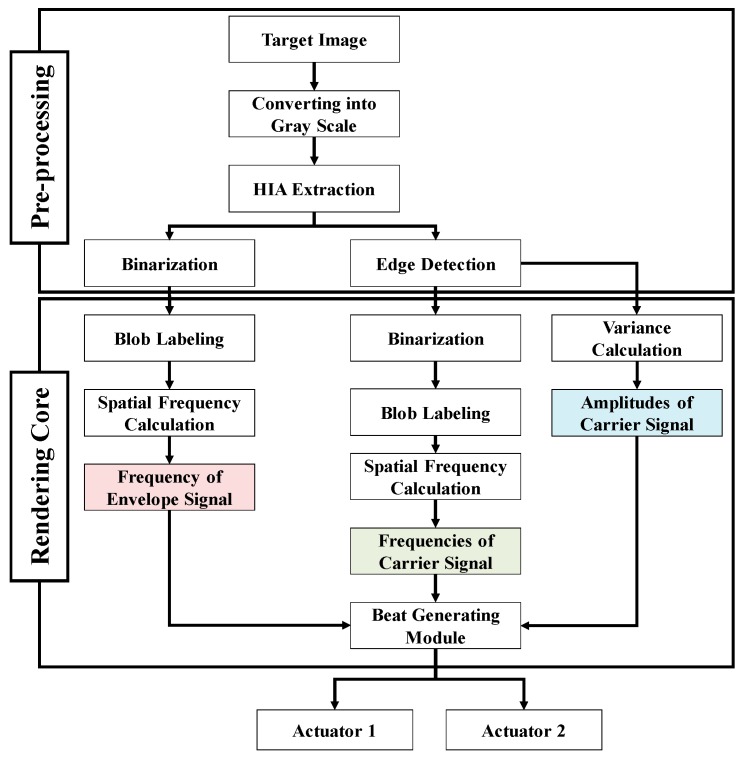
Procedure for the proposed rendering method.

The aim of our rendering method was to haptically simulate a target object’s surface (consisting of a micro- and macro-textures). To simulate this surface haptically, the proposed rendering method uses a beat vibration with two frequencies (beat frequency and carrier frequency). The macro- and micro-textures of the surface are simulated by the beat and carrier frequencies, respectively. This method is composed of a preprocessing module and rendering core module as shown in Figure 2. The preprocessing module converts the target surface into two images for extracting beat frequency and carrier frequency. The rendering core computes the beat frequency (envelope frequency) and the carrier frequency.

### 3.1. Preprocessing Procedure

**Figure 3:**
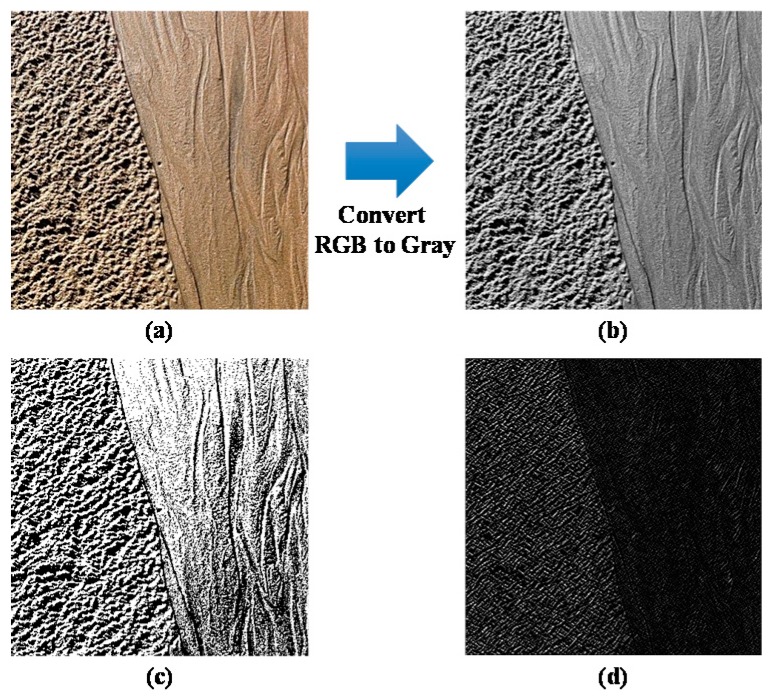
Converting RGB to gray value (**a**) target image, and (**b**) gray scale image; and tactile images, (**c**) binary image, and (**d**) edge detected image.

Commonly, the probe of a haptic device is modeled as a point, called the haptic interface point. Whenever a user explores a virtual environment with their finger, the contact portion between the finger and a target surface is not a point, but an area. This area is defined as the haptic interface area (HIA) [31]. To extract the haptic information from a target surface, we first conducted an image preprocessing. Here, the target surface image (Figure 3a) was converted into a gray scale image (Figure 3b). Then, the image binarization was performed to convert the grayscale image into a binary image, which only has two colors (white and black) as shown in Figure 3c. White and black areas in the binary image are regarded as the macro-texture of the target object’s surface. The frequency of the micro texture of the target object’s surface is determined from the edge-detected image, which consists of many high-frequency components. To detect the edges of the target image, the Sobel edge detection algorithm was adopted on the grayscale image, as shown in Figure 3d.

### 3.2. Determining the Beat Frequency

It is important to extract the haptic signal from a binary image, because it can render the macro-texture of a target surface. As mentioned before, the macro-texture of a surface can be represented by the beat frequency. Therefore, we first computed the HIA and then labeled black blobs. As it is not easy for humans to sense blobs smaller than the spatial resolution of the Merkel’s disks (or the Meissner corpuscles) [32], we have excluded the blobs smaller than the receptive field. Since the mean receptive field area of the Meissner corpuscle is generally known as 12.6 mm^2^ [33], we excluded the blobs smaller than 12.6 mm^2^ in this work.

**Figure 4:**
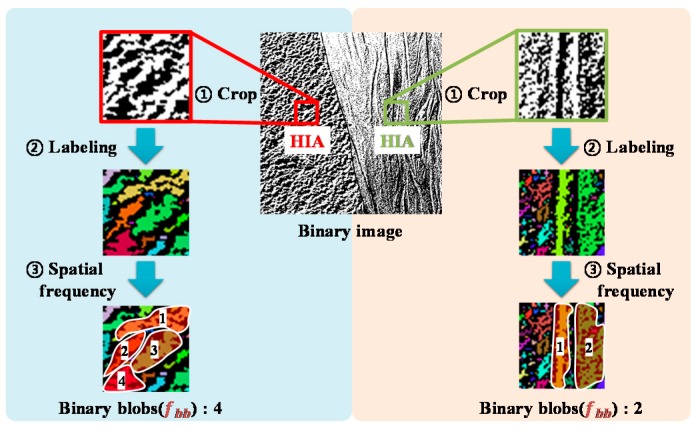
Examples of determining the beat frequency.

Let us consider a binary image that has been extracted from a nonhomogeneous surface with two regions of different roughness, as shown in Figure 4. After labeling all the blobs in the image, except the blobs smaller than the spatial resolution of the Merkel’s disk (or the Meissner corpuscles), we counted all the valid blobs in the HIA. We defined the number of the valid blobs as the spatial frequency of the HIA. When a user touches the left portion of the target surface, the spatial frequency of the HIA is four, and when interacting with the right portion, the spatial frequency of the HIA is two. This spatial frequency was then normalized by the number of maximum valid blobs (which can stimulate the human finger) in the HIA. The beat frequency was computed by multiplying this normalized value with the frequency bandwidth (0.3~40 Hz) of the Merkel’s disk and Meissner corpuscles [34,35], and can be expressed by the following equation:

(1) $$f_{b}=\;\alpha \;\left\{f_{bb}\;\left(f_{h}-\;f_{l}\right)+f_{l}\right\}$$

where $f_{b}$ is the beat frequency; α is a scale factor; $f_{bb}$ is the normalized spatial frequency of the binary blobs; $f_{h}$ is the highest frequency in the bandwidth of the Merkel’s disk and the Meissner corpuscles; and $f_{l}$ is the lowest frequency in the bandwidth of the Merkel’s disk and the Meissner corpuscles.

### 3.3. Determining the Carrier Frequency and Amplitude

**Figure 5:**
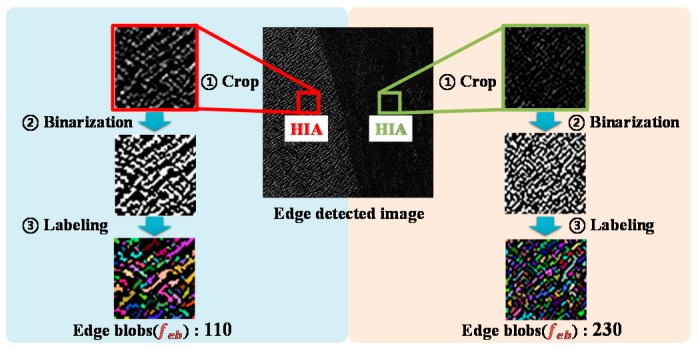
Examples of determining the carrier frequency.

The spatial frequency of the edge-detected image was computed by counting the number of edge blobs, and was then used for determining the carrier frequency of the beat wave. To extract the carrier frequency, we converted the edge-detected image to a binary image first. Then, we labeled the white blobs and calculated their spatial frequency in the HIA. If the spatial frequency increases, we can regard the target surface as smooth. From Figure 5, it is evident that the right portion of the image is smoother than the left portion. The spatial frequency was normalized by the number of maximum valid edge blobs in the HIA. Then, we computed the carrier frequency using this normalized value within the bandwidth of the Pacinian corpuscle (40~400 Hz) [34,35], as shown in the following equation:

(2) $$f_{c}=\;\alpha \;\left\{f_{eb}\;\left(f_{ph}-\;f_{pl}\right)+f_{pl}\right\}$$

where $f_{c}$ is the carrier frequency; α is a scale factor; $f_{eb}$ is the normalized spatial frequency of the edge blobs; $f_{ph}$ is the highest frequency in the bandwidth of the Pacinian corpuscles; and $f_{pl}$ is the lowest frequency in the bandwidth of the Pacinian corpuscles.

**Figure 6:**
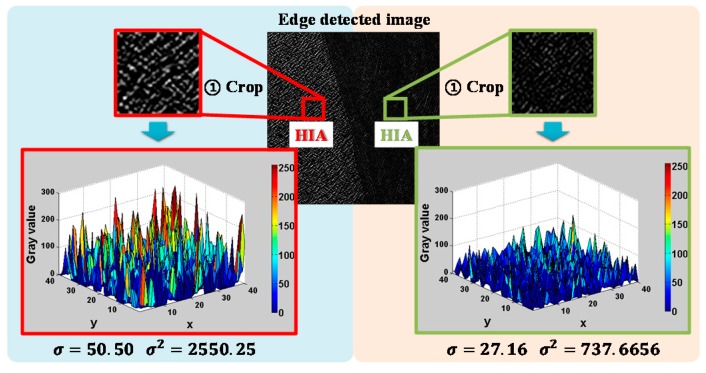
Examples of calculating the parameter (variance) required for the calculation of the amplitude of the carrier wave.

The amplitude of the carrier wave is also an important factor to haptically simulate the surface of the target object. To calculate the amplitude of the carrier wave, we used the variance, which is proportional to the variation in the height of the target surface. As shown in Figure 6, we computed the variance of the grayscale values in the HIA, and it is evident that the variance of the left portion is larger than that of the right portion. Thus, the variance was used as a parameter to compute the amplitude of the carrier wave, as shown in the following Equation:

(3) $$A_{c}=\;1-\;\frac{1}{1+\;\alpha \sigma^{2}}$$

where $A_{c}$ is the normalized amplitude of the carrier wave, α is a scale factor, and $\sigma^{2}$ is the variance of the grayscale values in the HIA.

By using these three equations (Equations (1)–(3)), we can determine the three major parameters (the beat frequency, the carrier frequency, and its amplitude) to haptically render a target surface consisting of macro/micro textures.

## 4. Experiments and Results

### 4.1. Experimental Environment

**Figure 7:**
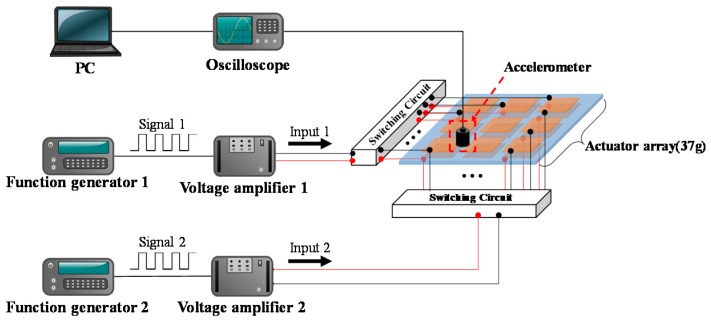
Experimental environment to measure the vibrational acceleration of the actuator array.

To investigate the effectiveness of the proposed haptic rendering of the flexible and transparent film-type vibrotactile actuator array, we constructed an experimental environment consisting of function generators, voltage amplifiers, an oscilloscope, a PC, an accelerometer, and a flexible and transparent haptic actuator array as shown in Figure 7. The flexible and transparent haptic actuator array was manufactured using a piezoelectric polymer (PVDF) with transparent and flexible electrode patterns and was mounted onto a thin acrylic plate. Two switching circuits, which are composed of infrared LEDs (light-emitting diode) and opto-diodes, were connected to each actuator cell. High-voltage and high-frequency switching was achieved by using high voltage opto-diode (OZ100SG, Voltage Multipliers Inc., Visalia, CA, USA), which can be activated by infrared signal. Two function generators (Protek 9305, GSInstech, Incheon, Korea) and two voltage amplifiers (Trek 10/40A-HS, Trek Inc., Lockport, NY, USA) were used for generating the beat vibration. An accelerometer (Charge accelerometer type 4393, Brüel & Kjær, Nærum, Denmark) was attached onto a portion between the two adjacent cells. The measured vibration dataset was conveyed to an oscilloscope (MSO/DPO 2000, Tektronix Inc., Beaverton, OR, USA) and was stored in a PC.

### 4.2. Experimental Results

**Figure 8:**
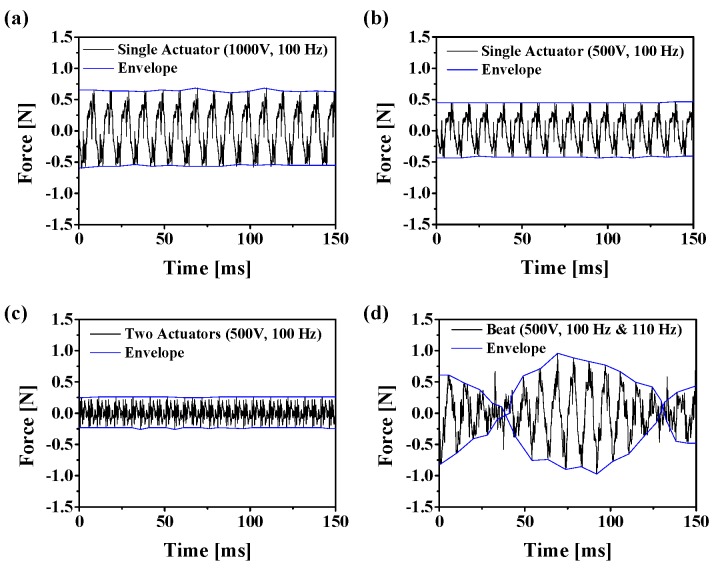
Results of the measured accelerations under four conditions; (**a**) 1000 V_pp_ (100 Hz) + 0 V, (**b**) 500 V_pp_ (100 Hz) + 0 V, (**c**) 500 V_pp_ (100 Hz) + 500 V_pp_ (100 Hz), and (**d**) 500 V_pp_ (100 Hz) + 500 V_pp_ (110 Hz).

We conducted an experiment with four input sets consisting of two AC sinusoidal input voltages: 1000 V_pp_ (100 Hz) + 0 V, 500 V_pp_ (100 Hz) + 0 V, 500 V_pp_ (100 Hz) + 500 V_pp_ (100 Hz), and 500 V_pp_ (100 Hz) + 500 V_pp_ (110 Hz). Figure 8 shows the haptic behavior of the proposed haptic actuator array under the four input sets. As expected, the vibration amplitude of the developed transparent flexible actuator was proportional to the input voltage. Let us take 500 V_pp_ (100 Hz) + 500 V_pp_ (110 Hz) as an example. Although the input frequencies were 100 Hz and 110 Hz, the rendered signal became the beat vibration with two frequencies (beat frequency (10 Hz) and carrier frequency (105 Hz)). This means that the rendered vibration could stimulate both Meissner corpuscle and Pacinian corpuscle simultaneously. In addition, although the input voltage was 500 V, this actuator could create large vibration amplitude without considering the phase control.

### 4.3. Performance Evaluation

**Figure 9:**
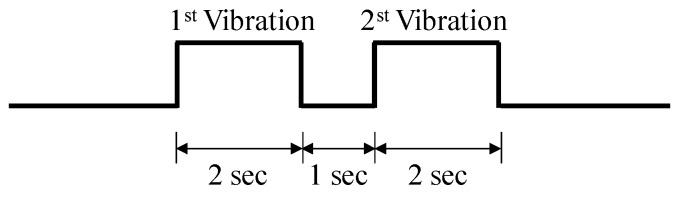
Time chart of stimulation.

The objective of this study was to haptically render a virtual object’s surface, consisting of a macro/micro texture, with beat vibration. Therefore, we need to investigate whether human operators can recognize and discriminate the two vibrational frequencies. To verify this, we conducted three experiments. Ten subjects aged between 23 and 30 years (average age: 26.5 years) participated. Among the subjects, seven subjects were male and three were female. Pink noise was provided to the subjects to exclude the vibration sounds from the actuator array during all experiments. Two vibrational stimuli (2 s long) were conveyed to the subjects with a 1 s pause between the two stimuli, as shown in Figure 9.

The first and second vibrations shown in Figure 9 were selected as 100 Hz or 105 Hz, and they could be the same or different. We prepared four sets of the vibrations, as shown in Table 1. We then observed whether the subjects could distinguish the 1st vibration and the 2nd vibration. The subjects were provided with a randomly selected set among the prepared four sets. Each of them answered whether the two vibratory stimuli are “same” or “different”. In the experiment, each subject experienced every set. To enhance the reliability, this procedure was repeated 10 times. Because the number of the subjects was 10, the total number of the trials was 40 per person. Table 2 shows the result of the experiment. For set 2 and set 3, the correct answer percentage were below 30%. The average of the correct answer rates was 48% (SD: 9.33). Therefore, this result shows that the participants could not distinguish between the two different frequencies.

We conducted another experiment to show that the users could discriminate between the beat vibration and pure vibration (vibration without beat effect). The subjects from the previous experiment participated in this test. The subjects experienced a standard vibration (beat vibration: 100 Hz + 105 Hz), and a comparison vibration (100 Hz + 100 Hz), randomly. Then, they compared the standard vibration (beat vibration) and comparison vibration. We prepared four sets of these vibrations as shown in Table 3. During the experiment, each subject experienced the first vibration for 2 s, after which there was a pause for 1 s, and then experienced the second vibration for 2 s, as shown in Figure 9. Four sets were presented to the subjects in a random order. After experiencing each set, the subjects answered whether the vibrations were “same” or “different”. In the experiment, each subject experienced every set. Each subject was provided a pink sound noise to prevent the subject from taking a guess due to the vibration sound, as with the previous experiment. To enhance the reliability, this procedure was repeated 10 times for each set. Table 4 shows the correct answer rates of the subjects. The correct answer percentage for set 4 was the lowest (84%) and the average of the correct answer rates was 90.5% (SD: 8.56). Therefore, this result shows that the subjects could perceive the beat vibration and could clearly distinguish between the beat vibration and the pure vibration (vibration without beat).

**Figure 10:**
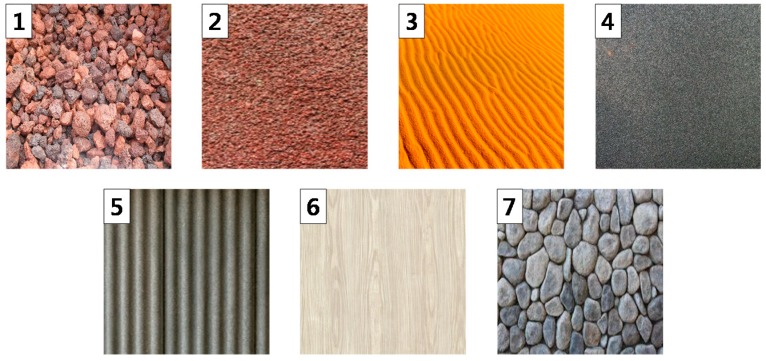
Seven prepared images for the third experiment.

Another experiment was conducted to show that the proposed rendering method could haptically simulate a variety of virtual objects’ surfaces. As shown in Figure 10, seven images were prepared in this experiment. We then printed the seven images onto papers. Each sheet of the paper contained one image each. First, the subject rubbed the touch panel with a soft vibrotactile actuator array using their finger without watching the virtual object, and then watched the printed images. Subsequently, the subject selected the printed image, which they thought was the same as the haptically sensed image. In the experiment, each subject experienced every image.

Each stimulus was created 10 times. The results of all participants were used to form a confusion matrix (Table 5). The rows of the confusion matrix represent the images presented to the participants via tactile stimulation, and the columns represent the images selected by the participants. In the case of the three images (1, 6, and 7), the accuracy was over 90% and the correct answer percentage for the rest were over 80%. The lowest correct answer percentage was 81%. From the results, it is evident that the subjects were not only able to distinguish one image from another, but also represent the graphical images haptically.

## 5. Conclusions

This paper proposes a haptic rendering method based on the beat phenomenon, which does not consider the vibration phase, for flexible EAP actuators array. The proposed rendering method is composed of a preprocessing module and a rendering core module. The preprocessing module converts a target surface into a binary image and an edge-detected image to obtain the basic components of the haptic information. The rendering core module computes the beat frequency from the binary image and carrier frequency from the edge detected image. Furthermore, the rendering core module calculates the amplitude of the carrier signal from the edge-detected image. The computed frequencies and the amplitude of the beat wave were used to haptically render a target object’s surface. Experiments were conducted to investigate the feasibility of the proposed rendering method. The results showed that the proposed rendering method could haptically simulate various objects’ surfaces without any phase control. We expect that the proposed haptic rendering method could be used for not only improving the performance of next generation flexible and soft devices, but also immersive interaction with a target object in a virtual environment.

## Figures and Tables

**Table 1:** Patterns and the expected answers of the first experiment.

	1st Vibration	2nd Vibration	Expectation
set 1	100 Hz	100 Hz	“Same”
set 2	100 Hz	105 Hz	“Different”
set 3	105 Hz	100 Hz	“Different”
set 4	105 Hz	105 Hz	“Same”

**Table 2:** The result of the first experiment.

		Presented			
		1	2	3	4
**Response**	**“Same”**	**75**	80	71	**68**
	**“Different”**	25	**20**	**29**	32

**Table 3:** Patterns of the second experiment.

	1st Vibration	2nd Vibration
set 1	100 Hz + 100 Hz	100 Hz + 100 Hz
set 2	100 Hz + 100 Hz	100 Hz + 105 Hz
set 3	100 Hz + 105 Hz	100 Hz + 100 Hz
set 4	100 Hz + 105 Hz	100 Hz + 105 Hz

**Table 4:** The result of the second experiment.

		Presented			
		1	2	3	4
**Response**	**“Same”**	**96**	6	12	**84**
	**“Different”**	4	**94**	**88**	16

**Table 5:** Confusion matrix of the third experiment’s results.

		Presented						
		1	2	3	4	5	6	7
**Response**	**1**	**95**	1		**2**			
	**2**	2	**86**	9	2	4		1
	**3**		**9**	**84**	1	7	1	
	**4**	1	**3**	**2**	88	4	1	1
	**5**	2	**1**	**4**	3	81	5	
	**6**			**1**	2	3	91	
	**7**				2	1	2	98
